# Inspire: A New Effort on Geroscience in Toulouse, France

**DOI:** 10.1093/geroni/igab046.1159

**Published:** 2021-12-17

**Authors:** Felipe Sierra, Bruno Vellas

**Affiliations:** 1 CHU Toulouse, France, Toulouse, Midi-Pyrenees, France; 2 CHU Toulouse: Centre Hospitalier Universitaire de Toulouse, Gérontopôle de Toulouse, Institut du Vieillissement, Midi-Pyrenees, France

## Abstract

The Inspire project of the Toulouse Hospital System is a comprehensive approach to health care in older adults, focused on maintenance of health and physical function. At the core of the project are human, mouse and killifish cohorts, which in the case of humans, is comprised of 1,000 subjects of ages 20 and above, which are followed for a total of 10 years, both via visits to the clinic, and electronic follow-up via the ICOPE app. At recruitment they are stratified as robust, pre-frail or frail according to Fried’s criteria, and then followed for loss of Intrinsic Capacities, as defined by WHO. A parallel cohort of Swiss mice with enhanced (exercise) and decreased (high fat diet) health will be used to measure concordant parameters. The project is generating a significant biorepository that is being used to pursue research in several areas where Toulouse has a significant research strength.

